# Co-Operation in Preparing Mouths for Dentures

**Published:** 1921-06

**Authors:** Frank Mihos


### CO-OPERATION IN PREPARING MOUTHS FOR DENTURES

BY FRANK MIHNOS, D.M.D.

The same exact, or standardized technique can not be used in all cases in preparing mouths for dentures, be they partial or complete, for several reasons. Every prosthodontist or practitioner has a different idea of the way he wishes a mouth to look for receiving his ideal work. Some men do not care for ridges, others want plenty of ridge, some wish the anterior plates taken off and some want them left full. So you see there are many ideas to contend with from the exodontist's point of view.

The exodontist has been ordered to remove the patient's teeth. He does so, and we will suppose that there is considerable destruction because of infected areas which were removed. The patient then goes back to the man who has charge of him. This man looks over the mouth and wonders, for the results are not what he expected and immediately the exodontist is condemned, for, in this man's opinion he has ruined the patient's mouth.

Now dentistry is far from an exact science, but it is probably the most exact of the medical sciences, and the X-ray has probably done the most toward advancing it to its high position today. By the X-ray we can make approximately 65 to 85 per cent of our diagnoses. Therefore our first step in proceeding is to get a radiogram of the teeth to be removed and their surrounding tissues.

The prosthodontist or practitioner then takes an impression of the mouth and pours a model. This model may now be trimmed and cut down, giving him a picture of the mouth in what he thinks is an ideal condition for his work. The two men co-operate in this way. The prosthetist or practitioner with his model shows the exodontist just how he wishes the mouth left after the teeth are removed. The exodontist with the radiogram knows where there will, or will not be necessary destruction of tissue. Perhaps it will change the model a bit, but between the two after every detail has been studied, a mutual working plan can be agreed upon whereby a very satisfactory result can be attained.

The operation is now performed and the mouth is shaped up to represent the model. For example, if there is a pronounced fullness or protrusion with a large ridge a buccal resection is made and the desired amount of tissue is taken off to correspond with the model.

If there is only a little too much ridge and a buccal resection would be too severe, then the teeth may be carefully removed with the forceps and a V-shaped trough made in the center of the ridge, the buccal plate being then compressed and sutured.

If the mouth will not stand loss of tissue then after the careful removal of the teeth, the very sharpest edges of bone are smoothed or rounded, leaving the mouth as near the ideal state as possible.

While every case presents a problem of its own, yet the results obtained by the exodontist who may modify the operation according to the model will be surprising.

In the past the exodontist was instructed to take out the teeth and trim up the ridge. From the standpoint of a surgical operation he probably was a bit radical in cutting down the buccal plate so that he would have tissue enough to be brought together so that it might heal by first intention. The immediate results are very fine, but it has left us facing a very grave proposition. The operation was too radical and today we find men who are turning prematurely gray from worry, for some of their patients have been left with mouths which will not carry dentures.

In some cases where we have plenty of tissue the wound may heal by first intention and the impression can be taken for permanent dentures within five to ten days. Those cases are few. Most mouths demand that we keep most of the tissue in as near a normal condition as possible and will be in better condition at the end of five years if done in a conservative manner.

It is true we are living in a very progressive age. There is a decided tendency to do our work as rapidly as possible, but the inevitable fact remains that we can not, with impunity, drive nature's processes. Therefore we must decide on a conservative course and this can only be done by the closest and most careful co-operation of practitioner, exodontist and patient.—*Northwestern Journal*, 919 Selling Bldg.

